# Contribution of Sex Differences to HIV Immunology, Pathogenesis, and Cure Approaches

**DOI:** 10.3389/fimmu.2022.905773

**Published:** 2022-05-25

**Authors:** Jose A. Moran, Shireen R. Turner, Matthew D. Marsden

**Affiliations:** ^1^Department of Microbiology and Molecular Genetics, School of Medicine, University of California, Irvine, CA, United States; ^2^Department of Medicine (Division of Infectious Diseases), School of Medicine, University of California, Irvine, CA, United States

**Keywords:** HIV, sex differences, pathogenesis, persistence, latency, cure, sexual dimorphism

## Abstract

Approximately 38 million people were living with human immunodeficiency virus (HIV) in 2020 and 53% of those infected were female. A variety of virological and immunological sex-associated differences (sexual dimorphism) in HIV infection have been recognized in males versus females. Social, behavioral, and societal influences play an important role in how the HIV pandemic has affected men and women differently. However, biological factors including anatomical, physiologic, hormonal, and genetic differences in sex chromosomes can each contribute to the distinct characteristics of HIV infection observed in males versus females. One striking example of this is the tendency for women to have lower HIV plasma viral loads than their male counterparts early in infection, though both progress to AIDS at similar rates. Sex differences in acquisition of HIV, innate and adaptive anti-HIV immune responses, efficacy/suitability of specific antiretroviral drugs, and viral pathogenesis have all been identified. Sex differences also have the potential to affect viral persistence, latency, and cure approaches. In this brief review, we summarize the major biological male/female sex differences in HIV infection and their importance to viral acquisition, pathogenesis, treatment, and cure efforts.

## Introduction

Human immunodeficiency virus (HIV) is a retrovirus that causes acquired immunodeficiency syndrome (AIDS) by infecting several important immune cell types including CD4+ T lymphocytes and macrophages (1, 2). Over 50% of people infected worldwide with HIV are female (3). While depletion of CD4+ T cells and progression to AIDS occurs in both males and females, there are significant sex-related differences in the course of infection. These “sex differences” have a biological origin, including hormonal or genetic. This is distinct from gender differences, which generally have a social or behavioral origin, including gendered socio-economic inequities such as unequal access to healthcare, financial resources, and education (4–8). While both sex and gender differences are important in understanding the HIV pandemic, this review is focused on biological sex differences that affect HIV infection.

Sexual dimorphism in the mammalian immune system has long been recognized in the context of infection with many different pathogens, including influenza viruses, hepatitis B virus, and the parasite *Entamoeba histolytica* (9–11). Females often have more effective immune responses against pathogens but are also more prone to autoimmune diseases such as multiple sclerosis, scleroderma, and systemic lupus erythematosus (9, 12, 13). Many important aspects of the mammalian innate and adaptive immune response have been identified to vary in a sex-dependent manner, including the activity of key immune-related signal transduction pathways, the relative numbers of T cells, B cells and immunoglobulins, and the activation state of different immune cell subsets (9). Of particular interest, in the context of HIV, are the higher CD4+ T cell counts observed in both females compared to males. Females also have higher CD4/CD8 ratios and their macrophages have higher phagocytic activity and activation states than those from males (9). Therefore, in addition to broad immunological effects, sex differences directly influence the preferred target cells for HIV infection.

**Figure 1:**
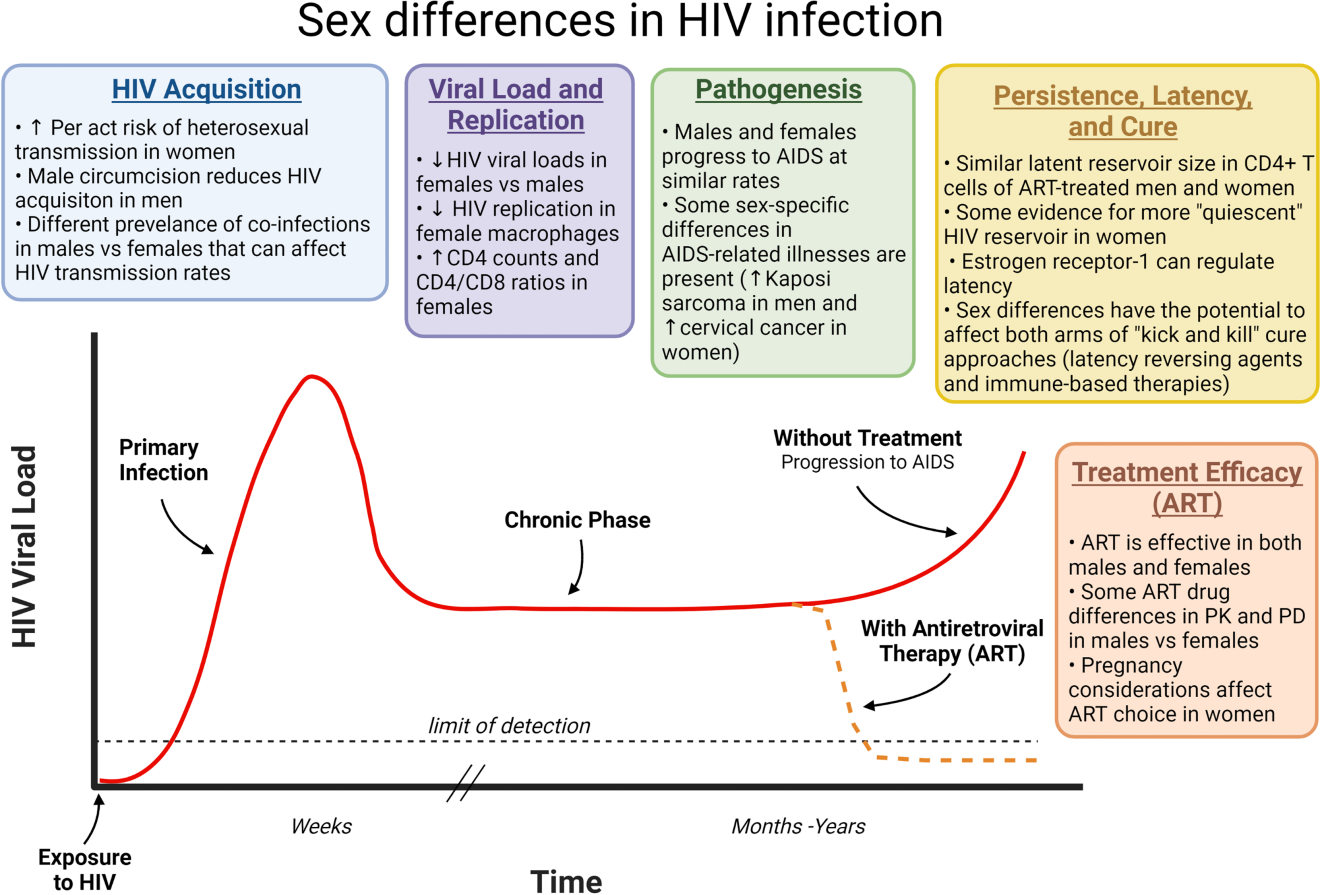
How sex differences can affect different phases of HIV infection. HIV infection progresses from primary infection through a chronic phase that often lasts years before development of AIDS approximately 10 years after HIV acquisition. ART prevents this disease progression by inhibiting virus replication and reducing viral loads. Sex differences can affect each phase of this infection as indicated. ART, Antiretroviral therapy; HIV, human immunodeficiency virus; AIDS, acquired immunodeficiency syndrome; PK, pharmacokinetics; PD, Pharmacodynamics.

Sex differences in HIV infection have largely been attributed to anatomical, physiologic, hormonal, and genetic differences, including during pre- and post-adolescence, and in old age (14). This review will provide a brief overview of the main ways in which sex-based differences can affect HIV infection, including during initial acquisition, subsequent viral replication, pathogenesis, treatment, and viral reservoirs/cure approaches (**Figure 1**).

### Sex Differences in Acquisition of HIV

With the widespread availability of ART, advent of pre-exposure prophylaxis (PrEP) and the continued education of HIV transmission risks, rates of HIV infection in the U.S. are declining (15). In 2019, the Center for Disease Control and Prevention (CDC) reported 19% of new HIV diagnoses in the US to be women (16), and worldwide over half of HIV infected people are female (6). Heterosexual HIV transmission is the main mode through which women become HIV-infected (84%) (16). Generally, receptive partners are at greater risk of contracting HIV during sexual intercourse due to large viral loads present in pre-seminal and seminal fluids from untreated HIV-infected insertive partners (17). Hence, transmission of HIV to women is more likely than the reverse during heterosexual intercourse when considering per-sexual-act risk. In higher-income countries, in the absence of antiretroviral therapy (ART), male-to-female transmission (0.08%) is double that of female-to-male (0.04%). In lower-income countries per-act male-to-female transmission (0.38%) was also higher than the reverse (0.30%) though this excludes transfer in commercial sex work (18). A similar trend is observed with insertive (0.17%) and receptive (1.25%) anal intercourse per-act risk, which may also be fueling heterosexual transmission (19).

HIV infects cells by binding to the CD4 receptor and a coreceptor (most commonly CCR5 or CXCR4) (20). CCR5 surface expression on CD4+ T cells is higher in men than women, potentially contributing to differences in HIV viral load in infected individuals (21). Langerhans cells are present in the vaginal mucosa and foreskin of uncircumcised men and have been shown to transport HIV to CD4+ T cells without becoming infected themselves (22–25). Hence, to lower heterosexual transmission, there has been a promotion of male circumcision, which reduces HIV acquisition in men by approximately 60% *via* several mechanisms including preventing the loss of epithelial barrier integrity and reducing the number of Langerhans cells present to transport the virus to T cells in draining lymph nodes (26–28).

Hormonal changes during menstruation have drastic effects on vaginal tissue physiology and immunologic characteristics, and on the vaginal microbiome (9, 29). Estrogen is protective against simian immunodeficiency virus (SIV) infection in rhesus macaques, though estrogen seems to have a more complex concentration-dependent role on inflammation in humans (30–32). At higher levels, estrogen is thought to be associated with lower HIV acquisition due to a decrease in migration of T cells and macrophages, and pro-inflammatory signal regulation (32, 33). Use of certain contraceptives, including injectable progestin-only contraceptives in women have also been associated with increased numbers of HIV target cells (CCR5+ CD4 T cells) in the cervix and an increased risk of HIV acquisition (34).

Systematic, meta-analysis, and longitudinal studies have shown that previous or concurrent infections with certain pathogens, including herpes simplex virus type two (HSV-2), *Trichomonas vaginalis*, *Chlamydia trachomatis*, *Neisseria gonorrhoeae*, human papillomavirus (HPV), or general vaginal dysbiosis, can increase the risk of HIV acquisition (35–43). This is in part due to damage to the epithelia and mucosa caused by pre-existing infections, and also the associated immune inflammation, which increases the number of activated CD4+ T cells and macrophages present (the preferred host cells for HIV) (44). Many sexually transmitted infections increase HIV infection risk in both men and women, but some are more prevalent or affect one sex differently from the other. For example, *Trichomonas vaginalis* is 10-fold more prevalent and highly symptomatic in women but is largely asymptomatic (70%) in men (45). HSV-2 is also more prevalent in women, though HIV acquisition risk in HSV-2 infected women was comparable to men (37, 46, 47).

Development of a prophylactic vaccine to provide long-term protection from HIV infection or a therapeutic vaccine to suppress virus and prevent disease progression without ART is a major focus of the HIV field (48–50). Sexual dimorphism is also present in responses to vaccines, with women responding better to many vaccines, including influenza, hepatitis A, and hepatitis B (33, 51). Historically, females have been underrepresented in HIV vaccine trials which has complicated analysis of sex as a biological variable in these studies (52).

Together, these anatomic, physiologic, immunologic, and hormonal differences all contribute to observed differences in HIV acquisition dynamics in males and females.

## Sex Differences in HIV Viral Load and Replication

Sex-related differences in the mammalian immune system often involve more frequent and severe infections in males and more efficient immune responses in females (14). This trend is also evident in viral infections, including HIV infection (13). Sexual dimorphism in the immune system is in part associated with intrinsic genetic differences observed prior to adult maturation and therefore before strong hormonal influences are widely evident (14, 53). Females have higher CD4+ T cell counts than their male counterparts and also have higher CD4/CD8 ratios (9). In HIV-infected children, females were shown to have lower HIV RNA levels than males in untreated infection (53). Women are also reported to have at least 40% lower HIV RNA compared to their male counterparts, which is most evident earlier in the course of infection (4, 9, 54, 55). Interestingly, CD4+ T cells do not show sex-biased differences in their capacity to support HIV infection *in vitro* (56). Female primary monocyte-derived macrophages, however, were shown to be less susceptible to HIV infection *in vitro* than male primary macrophages. This is believed to be dependent on sex differences in host anti-HIV restriction factor SAMHD1 (56). Although the genes for HIV entry receptors are not located on sex chromosomes, many immune genes do lie on the X chromosome, including certain toll-like receptors (TLRs) (33). Specifically, TLR-7 and TLR-8 are present in the X-chromosome and can recognize single-stranded viral RNA (ssRNA), including HIV genomic RNA (33, 57). Due to the presence of two X-chromosomes in females, one is normally inactivated during the embryonic stage. However, errors in this process can occur, with reports of 15-23% of X-linked genes escaping inactivation, including TLR-7 (58, 59). Biallelic expression of important X-chromosome genes, like TLR-7, leads to higher protein levels and might therefore lead to a more robust innate immune response (33, 59, 60). In female plasmacytoid dendritic cells, TLR-7 activation with HIV ssRNA led to the production of higher levels of IFNα, which is commonly observed in the first week of HIV infection (61–63). It has also been demonstrated that at low concentrations, estradiol, an estrogen sex hormone, and progesterone affect HIV replication by increasing HIV transcription but at high concentrations they reduce HIV integration (64), although another study indicates that 17β-estradiol can inhibit HIV transcription by inducing a complex between β-catenin and estrogen receptor α on the HIV promoter (65). Hence, sex hormones can directly affect HIV replication, but their net effects can be complex and concentration dependent.

## Sex Differences in HIV Pathogenesis

During the long “asymptomatic” phase of HIV infection, high levels of virus replication and CD4+ T cell killing occur, but this is counterbalanced by the host immune system replenishing depleted CD4+ T cells *via* homeostatic proliferation. Eventually, this balance is lost and CD4+ T cell numbers decline to the point that effective immune responses against HIV and other pathogens can no longer be mounted, resulting in progression to AIDS (66). Women have higher CD4+ T cell counts, and lower HIV plasma RNA loads during the asymptomatic phase of infection, but both males and females progress to AIDS at similar rates (67–69). Females, however, progress to AIDS at higher rates when compared to males of equivalent HIV RNA load (67).

Examples of common infections during AIDS include candidiasis, both bacterial and *Pneumocystis jirovecii* pneumonia, cytomegalovirus (CMV), *Toxoplasma gondii* infections, and tuberculosis (70–72). Generally, these opportunistic infections do not have a sex-based bias to either male or female individuals (73, 74). Yet there are some exceptions, such as an increased risk of HSV-2 and toxoplasmosis infection in women (74–77). Certain viral pathogens can also cause cancer in immunodeficient individuals. One example is human herpesvirus-8 (HHV-8), also known as Kaposi sarcoma-associated herpesvirus (KSHV), which causes Kaposi sarcoma. There is a higher incidence of Kaposi sarcoma in men than women worldwide, and this trend is also evident when comparing HIV-infected men and women (78–80). HPV is common in both women and men but can cause cervical cancer at elevated rates in HIV-infected females (70, 74).

A very small subset of people are capable of naturally suppressing HIV to undetectable levels without ART treatment (elite controllers) (81, 82) or following cessation of ART (post-treatment controllers) (83). Women have been identified in some studies to be over-represented in each of these groups, which may be influenced by multiple genetic and immunologic factors but is broadly consistent with the enhanced immune responses against infectious diseases, including HIV, observed in females (9, 84–86).

In summary, differences in immune responses as well as exacerbation of infections and cancers that are also sex-biased in HIV uninfected individuals are features of HIV pathogenesis in males versus females.

## Sex Differences in HIV Treatment

Modern ART regimens effectively suppress plasma viral loads and prevent progression to AIDS in both males and females (87). However, in some cases the pharmacokinetic and pharmacodynamic profiles and side effects of ART differ between males and females (88–91). This is driven by differences in body weight, body fat content, extracellular water content, and many other factors that together can influence drug efficacy or side effects in men and women (92). Even among women, plasma concentrations of some antiretroviral drugs may vary during pregnancy or during different menstrual cycle phases (88). Consideration of potential teratotoxic effects on the gestating fetus and efficacy in preventing mother-to-child transmission of HIV when selecting an ART regimen are also specific to women (93–95). The immunologic profile of men and women can differ during ART. For example, after 48 weeks of combination ART qualitative and quantitative sex-associated differences in proinflammatory cytokines have been identified in plasma (96, 97).

Within the last decade, there have been major developments in a prophylactic approach to HIV infection. In 2012, the U.S. Food and Drug Administration (FDA) approved a daily tablet, which combined tenofovir disoproxil fumarate and emtricitabine as PrEP (98). This is effective in preventing infection in men and women (98–100). It does however take longer for women to achieve maximum protection for receptive vaginal intercourse (21 days) compared to receptive anal intercourse (7 days) due to lower concentrations of tenofovir in cervicovaginal tissues than in rectal tissues (101–103). The composition of the vaginal microbiome has also been shown to affect the efficacy of some PrEP approaches. For example, tenofovir gel was three times more effective at reducing HIV incidence in women with a vaginal bacterial community dominated by *Lactobacillus* versus those with *Gardnerella vaginalis*, potentially due to metabolism and inactivation of the drug by *Gardnerella vaginalis* (104). This may partially explain why PrEP with tenofovir was found to be more effective in men than women.

## Sex Differences in Persistence, Latency, and Cure Approaches

One of the primary reasons that HIV infection is not cured by ART alone is that HIV forms a latent reservoir in resting CD4+ T cells (105–109). These long-lived latently infected cells encode non-expressing HIV genomes in their chromosomes, which can reactivate to produce infectious virus years after they were initially infected. Depleting this latent reservoir is therefore a central goal of HIV cure efforts. However, the potential influence of biological sex has not been a major focus in HIV cure research, as evidenced by the significant underrepresentation of women in clinical HIV cure studies (52).

Several studies have evaluated the size of the latent reservoir in men versus women using different approaches. While total HIV DNA copy numbers in PBMC do not measure only activation-inducible, replication-competent, latent HIV reservoirs (due to the presence of defective or non-inducible proviruses) (110), HIV DNA levels at the time of treatment interruption has been shown to predict time-to-rebound upon cessation of ART in some cases (111). Cross-sectional studies have demonstrated lower HIV DNA copy numbers in PBMCs from women than men during ART (110, 112, 113). However, a prospective study of HIV-infected, ART-suppressed individuals consisting of 26 well-matched pairs of women and men did not show significant differences in the frequency of CD4+ T cells harboring total or integrated HIV DNA (114). Yet this study did identify increased HIV transcriptional activity in reservoir cells from men based on multiply spliced HIV transcripts and plasma viral loads measured by single-copy assays, suggesting the reservoir may be more quiescent in women. Another study investigated HIV reservoir size and other characteristics of resting CD4+ T cells from matched ART-suppressed men and women (22 of each sex) with a comprehensive collection of analyses, including a quantitative viral outgrowth assay, intact proviral DNA assay, and total HIV DNA measures (115). They did not find significant differences in the frequency of latent HIV in resting CD4+ T cells between men and women. Hence, if sex differences in the size of the replication-competent latent reservoir in CD4+ T cells are present, they do not appear to be large enough to readily detect in these relatively small study populations.

One potential approach for eliminating latently infected cells is a “kick and kill” strategy, which involves inducing expression of the latent HIV genome to allow the host cell to be killed by viral cytopathic effects or the host immune response (105). To accomplish this, latency reversal agents (LRAs) that reactivate HIV expression are currently being explored. Interestingly, using a primary cell *in vitro* model of HIV latency, it was found that there were not sex-based differences in latency reversal using five different compounds (ingenol-3,20-dibenzoate, bryostatin-1, 3-hydroxy-1,2,3-benzotriazin-4[3H]-one [HODHBt], Pam3CSK4, and vorinostat) (116). However, comprehensive studies of different LRA classes in *ex vivo* latently-infected cells from ART-suppressed patients or *in vivo* models have not yet been performed. An additional study has demonstrated that the estrogen receptor acts as a potent repressor of latency reversal (117). This study showed in several model systems that selective estrogen receptor modulators including tamoxifen, raloxifene, and fulvestrant can weakly induce proviral reactivation but, importantly, can also sensitize cells to reactivation with other LRAs, including IL-15, vorinostat, and TNFα. Using primary cells from well-matched men and women, they also found that the total inducible RNA reservoir was smaller in women than men. These data suggest that targeting estrogen receptor signaling may be useful in augmenting latency reversal and depleting reservoir cells (117). As observed for other drugs, differences between men and women in the pharmacokinetic and pharmacodynamic properties of individual or combination LRAs may also occur (4, 92). Proposed “kill” approaches to eliminate virus host cells after latency reversal include anti-HIV envelope antibody or immunotoxin therapeutics, augmentation of immune responses, and cytotoxic T cells bearing chimeric antigen receptors (105). This “kill” arm of the “kick and kill” therapy may also be affected by sex differences *via* the same mechanisms that distinguish male from female immune responses in untreated HIV infection (9).

There have been three reported cases of apparent HIV cure through therapeutic interventions. This includes the two well-documented cases of the “Berlin Patient” and “London Patient” who received allogeneic hemopoietic stem cell transplants with CCR5 Δ32 mutations during their treatment for leukemia or Hodgkin lymphoma, respectively (118–120). Although the “Berlin Patient” passed away in 2020 of cancer after 13 years of undetectable plasma viral loads without ART, the “London Patient” has remained HIV undetectable since cessation of ART in 2017 (119–121). In early 2022, the first female patient was reported to have been potentially cured of HIV following transplant with cord blood encoding a similar mutation CCR5 Δ32 for leukemia treatment. At that point she had undetectable viral loads for 14 months after discontinuing ART (118). The small number of these potential “cures” does not allow for systematic comparisons of sex differences, but they do provide a proof of concept for HIV cure in both sexes.

## Discussion

Intrinsic biological differences in males versus females can markedly influence the course of HIV infection (4, 5, 7). These sex-related factors affect acquisition of HIV, viral replication, pathogenesis, and the ART used in pre-exposure prophylaxis or treatment. The role of sex differences in HIV persistence and cure approaches is less well-defined. While there have been some important pioneering studies in this area, there is a pressing need to understand whether the characteristics or anatomic locations of persistent HIV reservoirs differ in males versus females during ART, and whether promising approaches for HIV cure behave similarly in both sexes. *In vivo* models (122–124) of HIV persistence and carefully planned clinical studies that specifically evaluate sex differences offer opportunities to achieve this goal and should be a focus of future work.

## Author Contributions

JM, ST, and MM all contributed to literature searches and writing of the manuscript. All authors contributed to the article and approved the submitted version.

## Funding

The author’s laboratory is supported by National Institutes of Health (NIH) grants P01AI131294 and R56AI124743. J.A.M. is a predoctoral trainee supported by U.S. Public Health Service training grant T32 AI007319 from the NIH.

## Conflict of Interest

The authors declare that the research was conducted in the absence of any commercial or financial relationships that could be construed as a potential conflict of interest.

## Publisher’s Note

All claims expressed in this article are solely those of the authors and do not necessarily represent those of their affiliated organizations, or those of the publisher, the editors and the reviewers. Any product that may be evaluated in this article, or claim that may be made by its manufacturer, is not guaranteed or endorsed by the publisher.

## References

[1] Wong ME Jaworowski A Hearps AC. The HIV Reservoir in Monocytes and Macrophages. Front Immunol (2019) 10:1435. doi: 10.3389/fimmu.2019.01435 31297114 PMC6607932

[2] Gottlieb MS Schroff R Schanker HM Weisman JD Fan PT Wolf RA. Pneumocystis Carinii Pneumonia and Mucosal Candidiasis in Previously Healthy Homosexual Men. N Engl J Med (1981) 305(24):1425–31. doi: 10.1056/nejm198112103052401 6272109

[3] UNAIDS. Global HIV & AIDS Statistics - Fact Sheet. Available at: https://www.unaids.org/en/resources/fact-sheet.

[4] Griesbeck M Scully E Altfeld M. Sex and Gender Differences in HIV-1 Infection. Clin Sci (2016) 130(16):1435–51. doi: 10.1042/cs20160112 27389589

[5] Scully EP. Hidden in Plain Sight: Sex and Gender in Global Pandemics. Curr Opin HIV AIDS (2021) 16(1):48–53. doi: 10.1097/coh.0000000000000661 33278160 PMC8162835

[6] Frank TD Carter A Jahagirdar D Biehl MH Douwes-Schultz D Larson SL. Global, Regional, and National Incidence, Prevalence, and Mortality of HIV, 1980–2017, and Forecasts to 2030, for 195 Countries and Territories: A Systematic Analysis for the Global Burden of Diseases, Injuries, and Risk Factors Study 2017. Lancet HIV (2019) 6(12):e831–59. doi: 10.1016/S2352-3018(19)30196-1 PMC6934077 31439534

[7] Breskin A Adimora AA Westreich D. Women and HIV in the United States. PloS One (2017) 12(2):e0172367. doi: 10.1371/journal.pone.0172367 28207818 PMC5313170

[8] Sohler NL Li X Cunningham CO. Gender Disparities in HIV Health Care Utilization Among the Severely Disadvantaged: Can We Determine the Reasons? AIDS Patient Care STDs (2009) 23(9):775–83. doi: 10.1089/apc.2009.0041 PMC2859765 19663745

[9] Klein SL Flanagan KL. Sex Differences in Immune Responses. Nat Rev Immunol (2016) 16(10):626–38. doi: 10.1038/nri.2016.90 27546235

[10] vom Steeg LG Klein SL. SeXX Matters in Infectious Disease Pathogenesis. PloS Pathog (2016) 12(2):e1005374. doi: 10.1371/journal.ppat.1005374 26891052 PMC4759457

[11] Gay L Melenotte C Lakbar I Mezouar S Devaux C Raoult D. Sexual Dimorphism and Gender in Infectious Diseases. Front Immunol (2021) 12:698121. doi: 10.3389/fimmu.2021.698121 34367158 PMC8339590

[12] Youness A Miquel C-H Guéry J-C. Escape From X Chromosome Inactivation and the Female Predominance in Autoimmune Diseases. Int J Mol Sci (2021) 22(3):1114. doi: 10.3390/ijms22031114 33498655 PMC7865432

[13] Jacobsen H Klein SL. Sex Differences in Immunity to Viral Infections. Front Immunol (2021) 12:720952. doi: 10.3389/fimmu.2021.720952 34531867 PMC8438138

[14] Jaillon S Berthenet K Garlanda C. Sexual Dimorphism in Innate Immunity. Clin Rev Allergy Immunol (2019) 56(3):308–21. doi: 10.1007/s12016-017-8648-x 28963611

[15] HIV.gov. Available at: https://www.HIV.gov/HIV-basics/overview/data-and-trends/statistics.

[16] CDC. HIV and Women: HIV Diagnoses. Available at: https://www.cdc.gov/HIV/group/gender/women/diagnoses.html.

[17] Gupta P Mellors J Kingsley L Riddler S Singh MK Schreiber S. High Viral Load in Semen of Human Immunodeficiency Virus Type 1-Infected Men at All Stages of Disease and Its Reduction by Therapy With Protease and Nonnucleoside Reverse Transcriptase Inhibitors. J Virol (1997) 71(8):6271–5. doi: 10.1128/jvi.71.8.6271-6275.1997 PMC191898 9223532

[18] Boily M-C Baggaley RF Wang L Masse B White RG Hayes RJ. Heterosexual Risk of HIV-1 Infection Per Sexual Act: Systematic Review and Meta-Analysis of Observational Studies. Lancet Infect Dis (2009) 9(2):118–29. doi: 10.1016/S1473-3099(09)70021-0 PMC4467783 19179227

[19] Baggaley RF Owen BN Silhol R Elmes J Anton P McGowan I. Does Per-Act HIV-1 Transmission Risk Through Anal Sex Vary by Gender? An Updated Systematic Review and Meta-Analysis. Am J Reprod Immunol (2018) 80(5):e13039. doi: 10.1111/aji.13039 PMC6202169 30175479

[20] Berger EA Murphy PM Farber JM. Chemokine Receptors as HIV-1 Coreceptors: Roles in Viral Entry, Tropism, and Disease. Annu Rev Immunol (1999) 17(1):657–700. doi: 10.1146/annurev.immunol.17.1.657 10358771

[21] Portales P Clot J Corbeau P. Sex Differences in HIV-1 Viral Load Due to Sex Difference in CCR5 Expression. Ann Internal Med (2001) 134(1):81–2. doi: 10.7326/0003-4819-134-1-200101020-00023 11187428

[22] Ballweber L Robinson B Kreger A Fialkow M Lentz G McElrath MJ. Vaginal Langerhans Cells Nonproductively Transporting HIV-1 Mediate Infection of T Cells. J Virol (2011) 85(24):13443–7. doi: 10.1128/JVI.05615-11 PMC3233146 21976645

[23] Zhou Z Barry de Longchamps N Schmitt A Zerbib M Vacher-Lavenu M-C Bomsel M. HIV-1 Efficient Entry in Inner Foreskin Is Mediated by Elevated CCL5/Rantes That Recruits T Cells and Fuels Conjugate Formation With Langerhans Cells. PloS Pathog (2011) 7(6):e1002100–e. doi: 10.1371/journal.ppat.1002100 PMC3128116 21738469

[24] Esra RT Olivier AJ Passmore J-AS Jaspan HB Harryparsad R Gray CM. Does HIV Exploit the Inflammatory Milieu of the Male Genital Tract for Successful Infection? Front Immunol (2016) 7:245. doi: 10.3389/fimmu.2016.00245 27446076 PMC4919362

[25] Hussain LA Lehner T. Comparative Investigation of Langerhans' Cells and Potential Receptors for HIV in Oral, Genitourinary and Rectal Epithelia. Immunology (1995) 85(3):475–84. PMC1383923 7558138

[26] Krieger JN. Male Circumcision and HIV Infection Risk. World J Urol (2012) 30(1):3–13. doi: 10.1007/s00345-011-0696-x 21590467

[27] Gray RH Kigozi G Serwadda D Makumbi F Watya S Nalugoda F. Male Circumcision for HIV Prevention in Men in Rakai, Uganda: A Randomised Trial. Lancet (2007) 369(9562):657–66. doi: 10.1016/S0140-6736(07)60313-4 17321311

[28] Prodger JL Kaul R. The Biology of How Circumcision Reduces HIV Susceptibility: Broader Implications for the Prevention Field. AIDS Res Ther (2017) 14(1):49. doi: 10.1186/s12981-017-0167-6 28893286 PMC5594533

[29] Oertelt-Prigione S. Immunology and the Menstrual Cycle. Autoimmun Rev (2012) 11(6):A486–A92. doi: 10.1016/j.autrev.2011.11.023 22155200

[30] Smith SM Mefford M Sodora D Klase Z Singh M Alexander N. Topical Estrogen Protects Against SIV Vaginal Transmission Without Evidence of Systemic Effect. AIDS (2004) 18(12):1637–43. doi: 10.1097/01.AIDS.0000131393.76221.cc 15280774

[31] Smith SM Baskin GB Marx PA. Estrogen Protects Against Vaginal Transmission of Simian Immunodeficiency Virus. J Infect Dis (2000) 182(3):708–15. doi: 10.1086/315776 10950763

[32] Straub RH. The Complex Role of Estrogens in Inflammation. Endocr Rev (2007) 28(5):521–74. doi: 10.1210/er.2007-0001 17640948

[33] Fathi A Addo MM Dahlke C. Sex Differences in Immunity: Implications for the Development of Novel Vaccines Against Emerging Pathogens. Front Immunol (2021) 11:601170. doi: 10.3389/fimmu.2020.601170 33488596 PMC7820860

[34] Byrne EH Anahtar MN Cohen KE Moodley A Padavattan N Ismail N. Association Between Injectable Progestin-Only Contraceptives and HIV Acquisition and HIV Target Cell Frequency in the Female Genital Tract in South African Women: A Prospective Cohort Study. Lancet Infect Dis (2016) 16(4):441–8. doi: 10.1016/S1473-3099(15)00429-6 PMC5294917 26723758

[35] Masese L Baeten JM Richardson BA Bukusi E John-Stewart G Graham SM. Changes in the Contribution of Genital Tract Infections to HIV Acquisition Among Kenyan High-Risk Women From 1993 to 2012. AIDS (2015) 29(9):1077–85. doi: 10.1097/qad.0000000000000646 PMC4576156 26125141

[36] Masha SC Cools P Sanders EJ Vaneechoutte M Crucitti T. Trichomonas Vaginalis and HIV Infection Acquisition: A Systematic Review and Meta-Analysis. Sex Transm Infect (2019) 95(1):36–42. doi: 10.1136/sextrans-2018-053713 30341233 PMC6580735

[37] Looker KJ Elmes JAR Gottlieb SL Schiffer JT Vickerman P Turner KME. Effect of HSV-2 Infection on Subsequent HIV Acquisition: An Updated Systematic Review and Meta-Analysis. Lancet Infect Dis (2017) 17(12):1303–16. doi: 10.1016/S1473-3099(17)30405-X PMC5700807 28843576

[38] Atashili J Poole C Ndumbe PM Adimora AA Smith JS. Bacterial Vaginosis and HIV Acquisition: A Meta-Analysis of Published Studies. AIDS (Lond Engl) (2008) 22(12):1493–501. doi: 10.1097/QAD.0b013e3283021a37 PMC2788489 18614873

[39] McClelland RS Sangaré L Hassan WM Lavreys L Mandaliya K Kiarie J. Infection With Trichomonas Vaginalis Increases the Risk of HIV-1 Acquisition. J Infect Dis (2007) 195(5):698–702. doi: 10.1086/511278 17262712

[40] Barnabas RV Wasserheit JN Huang Y Janes H Morrow R Fuchs J. Impact of Herpes Simplex Virus Type 2 on HIV-1 Acquisition and Progression in an HIV Vaccine Trial (the Step Study). JAIDS J Acquired Immune Defic Syndr (2011) 57(3):238–44. doi: 10.1097/QAI.0b013e31821acb5 PMC3446850 21860356

[41] Malekinejad M Barker EK Merai R Lyles CM Bernstein KT Sipe TA. Risk of HIV Acquisition Among Men Who Have Sex With Men Infected With Bacterial Sexually Transmitted Infections: A Systematic Review and Meta-Analysis. Sex Transm Dis (2021) 48(10):e138–e48. doi: 10.1097/olq.0000000000001403 PMC8485981 33783414

[42] Houlihan CF Larke NL Watson-Jones D Smith-McCune KK Shiboski S Gravitt PE. Human Papillomavirus Infection and Increased Risk of HIV Acquisition. A Systematic Review and Meta-Analysis. AIDS (London England) (2012) 26(17):2211–22. doi: 10.1097/QAD.0b013e328358d908 PMC3831022 22874522

[43] Rositch AF Mao L Hudgens MG Moses S Agot K Backes DM. Risk of HIV Acquisition Among Circumcised and Uncircumcised Young Men With Penile Human Papillomavirus Infection. AIDS (London England) (2014) 28(5):745–52. doi: 10.1097/QAD.0000000000000092 PMC4074250 24149088

[44] de Jong MAWP de Witte L Oudhoff MJ Gringhuis SI Gallay P Geijtenbeek TBH. TNF-A and TLR Agonists Increase Susceptibility to HIV-1 Transmission by Human Langerhans Cells *Ex Vivo* . J Clin Invest (2008) 118(10):3440–52. doi: 10.1172/JCI34721 PMC2528910 18776939

[45] Bhakta SB Moran JA Mercer F. Neutrophil Interactions With the Sexually Transmitted Parasite Trichomonas Vaginalis: Implications for Immunity and Pathogenesis. Open Biol (2020) 10(9):200192–. doi: 10.1098/rsob.200192 PMC7536067 32873151

[46] Kaushic C Roth KL Anipindi V Xiu F. Increased Prevalence of Sexually Transmitted Viral Infections in Women: The Role of Female Sex Hormones in Regulating Susceptibility and Immune Responses. J Reprod Immunol (2011) 88(2):204–9. doi: 10.1016/j.jri.2010.12.004 21296427

[47] Freeman EE Weiss HA Glynn JR Cross PL Whitworth JA Hayes RJ. Herpes Simplex Virus 2 Infection Increases HIV Acquisition in Men and Women: Systematic Review and Meta-Analysis of Longitudinal Studies. AIDS (2006) 20(1):73–83. doi: 10.1097/01.AIDS.0000198081.09337.a7 16327322

[48] Burton DR Hangartner L. Broadly Neutralizing Antibodies to HIV and Their Role in Vaccine Design. Annu Rev Immunol (2016) 34(1):635–59. doi: 10.1146/annurev-immunol-041015-055515 PMC6034635 27168247

[49] Haynes BF Mascola JR. The Quest for an Antibody-Based HIV Vaccine. Immunol Rev (2017) 275(1):5–10. doi: 10.1111/imr.12517 28133795 PMC5384259

[50] Burton DR. Advancing an HIV Vaccine; Advancing Vaccinology. Nat Rev Immunol (2019) 19(2):77–8. doi: 10.1038/s41577-018-0103-6 PMC6425752 30560910

[51] Cook IF. Sexual Dimorphism of Humoral Immunity With Human Vaccines. Vaccine (2008) 26(29):3551–5. doi: 10.1016/j.vaccine.2008.04.054 18524433

[52] Curno MJ Rossi S Hodges-Mameletzis I Johnston R Price MA Heidari S. A Systematic Review of the Inclusion (or Exclusion) of Women in HIV Research: From Clinical Studies of Antiretrovirals and Vaccines to Cure Strategies. JAIDS J Acquir Immune Defic Syndr (2016) 71(2):181–8. doi: 10.1097/qai.0000000000000842 26361171

[53] Ruel TD Zanoni BC Ssewanyana I Cao H Havlir DV Kamya M. Sex Differences in HIV RNA Level and CD4 Cell Percentage During Childhood. Clin Infect Dis (2011) 53(6):592–9. doi: 10.1093/cid/cir484 PMC3160805 21840929

[54] Sharp PM Hahn BH. Origins of HIV and the AIDS Pandemic. Cold Spring Harb Perspect Med (2011) 1(1):a006841. doi: 10.1101/cshperspect.a006841 22229120 PMC3234451

[55] Romona DG Muhammad Jawad H Moien ABK Halla M Gulfaraz K. Global Epidemiology of HIV/AIDS: A Resurgence in North America and Europe. J Epidemiol Global Health (2021) 11(3):296–301. doi: 10.2991/jegh.k.210621.001 PMC8435868 34270183

[56] Szaniawski MA Spivak AM Bosque A Planelles V. Sex Influences SAMHD1 Activity and Susceptibility to Human Immunodeficiency Virus-1 in Primary Human Macrophages. J Infect Dis (2019) 219(5):777–85. doi: 10.1093/infdis/jiy583 PMC6376916 30299483

[57] Browne EP. The Role of Toll-Like Receptors in Retroviral Infection. Microorganisms (2020) 8(11):1787. doi: 10.3390/microorganisms8111787 PMC7697840 33202596

[58] Tukiainen T Villani A-C Yen A Rivas MA Marshall JL Satija R. Landscape of X Chromosome Inactivation Across Human Tissues. Nature (2017) 550(7675):244–8. doi: 10.1038/nature24265 PMC5685192 29022598

[59] Souyris M Cenac C Azar P Daviaud D Canivet A Grunenwald S. TLR7 Escapes X Chromosome Inactivation in Immune Cells. Sci Immunol (2018) 3(19):eaap8855. doi: 10.1126/sciimmunol.aap8855 29374079

[60] Souyris M Mejía JE Chaumeil J Guéry J-C. Female Predisposition to TLR7-Driven Autoimmunity: Gene Dosage and the Escape From X Chromosome Inactivation. Semin Immunopathol (2019) 41(2):153–64. doi: 10.1007/s00281-018-0712-y 30276444

[61] Meier A Chang JJ Chan ES Pollard RB Sidhu HK Kulkarni S. Sex Differences in the Toll-Like Receptor–Mediated Response of Plasmacytoid Dendritic Cells to HIV-1. Nat Med (2009) 15(8):955–9. doi: 10.1038/nm.2004 PMC2821111 19597505

[62] Utay NS Douek DC. Interferons and HIV Infection: The Good, the Bad, and the Ugly. Pathog Immun (2016) 1(1):107–16. doi: 10.20411/pai.v1i1.125 PMC4972494 27500281

[63] Guéry J-C. Sex Differences in Primary HIV Infection: Revisiting the Role of TLR7-Driven Type 1 IFN Production by Plasmacytoid Dendritic Cells in Women. Front Immunol (2021) 12:729233. doi: 10.3389/fimmu.2021.729233 34512664 PMC8432934

[64] Asin SN Heimberg AM Eszterhas SK. Estradiol and Progesterone Regulate HIV Type 1 Replication in Peripheral Blood Cells. AIDS Res Hum Retroviruses (2008) 24(5):701–16. doi: 10.1089/aid.2007.0108 18462082

[65] Szotek EL Narasipura SD Al-Harthi L. 17β-Estradiol Inhibits HIV-1 by Inducing a Complex Formation Between B-Catenin and Estrogen Receptor A on the HIV Promoter to Suppress HIV Transcription. Virology (2013) 443(2):375–83. doi: 10.1016/j.virol.2013.05.027 PMC3722310 23769242

[66] Douek DC Picker LJ Koup RA. T Cell Dynamics in HIV-1 Infection. Annu Rev Immunol (2003) 21(1):265–304. doi: 10.1146/annurev.immunol.21.120601.141053 12524385

[67] Farzadegan H Hoover DR Astemborski J Lyles CM Margolick JB Markham RB. Sex Differences in HIV-1 Viral Load and Progression to AIDS. Lancet (1998) 352(9139):1510–4. doi: 10.1016/S0140-6736(98)02372-1 9820299

[68] Prins M Robertson JR Brettle RP Aguado IH Broers B Boufassa F. Do Gender Differences in CD4 Cell Counts Matter? AIDS (1999) 13(17):2361–4. doi: 10.1097/00002030-199912030-00007 10597777

[69] Gandhi M Bacchetti P Miotti P Quinn TC Veronese F Greenblatt RM. Does Patient Sex Affect Human Immunodeficiency Virus Levels? Clin Infect Dis (2002) 35(3):313–22. doi: 10.1086/341249 12115098

[70] Geldmacher C Koup RA. Pathogen-Specific T Cell Depletion and Reactivation of Opportunistic Pathogens in HIV Infection. Trends Immunol (2012) 33(5):207–14. doi: 10.1016/j.it.2012.01.011 PMC3348406 22398371

[71] Shafran SD. Opportunistic Infections in HIV-Infected Patients. Can J Infect Dis (1992) 3(2):82–7. doi: 10.1155/1992/413713 PMC3328018 22529738

[72] Vergis EN Mellors JW. Natural History of HIV-1 Infection. Infect Dis Clinics North America (2000) 14(4):809–25. doi: 10.1016/S0891-5520(05)70135-5 11144640

[73] Kaplan JE Hanson D Dworkin MS Frederick T Bertolli J Lindegren ML. Epidemiology of Human Immunodeficiency Virus-Associated Opportunistic Infections in the United States in the Era of Highly Active Antiretroviral Therapy. Clin Infect Dis (2000) 30(Supplement_1):S5–14. doi: 10.1086/313843 10770911

[74] Gilad J Walfisch A Borer A Schlaeffer F. Gender Differences and Sex-Specific Manifestations Associated With Human Immunodeficiency Virus Infection in Women. Eur J Obstet Gynecol Reprod Biol (2003) 109(2):199–205. doi: 10.1016/S0301-2115(03)00048-4 12860342

[75] Phillips AN Antunes F Stergious G Ranki A Jensen GF Bentwich Z. A Sex Comparison of Rates of New AIDS-Defining Disease and Death in 2554 AIDS Cases. AIDS Europe Study Group AIDS (1994) 8(6):831–5. doi: 10.1097/00002030-199406000-00017 8086143

[76] Bernin H Lotter H. Sex Bias in the Outcome of Human Tropical Infectious Diseases: Influence of Steroid Hormones. J Infect Dis (2014) 209(suppl_3):S107–13. doi: 10.1093/infdis/jit610 24966190

[77] Dickson N Righarts A van Roode T Paul C Taylor J Cunningham AL. HSV-2 Incidence by Sex Over Four Age Periods to Age 38 in a Birth Cohort. Sex Transm Infect (2014) 90(3):243–5. doi: 10.1136/sextrans-2013-051235 24337730

[78] Cesarman E Damania B Krown SE Martin J Bower M Whitby D. Kaposi Sarcoma. Nat Rev Dis Primers (2019) 5(1):9. doi: 10.1038/s41572-019-0060-9 30705286 PMC6685213

[79] IeDEA TA-dCPWGf, EuroCoord Ci. Comparison of Kaposi Sarcoma Risk in Human Immunodeficiency Virus-Positive Adults Across 5 Continents: A Multiregional Multicohort Study. Clin Infect Dis (2017) 65(8):1316–26. doi: 10.1093/cid/cix480 PMC5850623 28531260

[80] Cooley TP Hirschhorn LR O'Keane JC. Kaposi's Sarcoma in Women With AIDS. AIDS (1996) 10(11):1221–5. doi: 10.1097/00002030-199609000-00007 8883583

[81] Blankson JN Siliciano RF. Elite Suppression of HIV-1 Replication. Immunity (2008) 29(6):845–7. doi: 10.1016/j.immuni.2008.12.002 19100698

[82] Carrington M Walker BD. Immunogenetics of Spontaneous Control of HIV. Annu Rev Med (2012) 63(1):131–45. doi: 10.1146/annurev-med-062909-130018 PMC3725592 22248321

[83] Li JZ Blankson JN. How Elite Controllers and Posttreatment Controllers Inform Our Search for an HIV-1 Cure. J Clin Invest (2021) 131(11):1–6. doi: 10.1172/JCI149414 PMC8159676 34060478

[84] Crowell TA Gebo KA Blankson JN Korthuis PT Yehia BR Rutstein RM. Hospitalization Rates and Reasons Among HIV Elite Controllers and Persons With Medically Controlled HIV Infection. J Infect Dis (2014) 211(11):1692–702. doi: 10.1093/infdis/jiu809 PMC4447832 25512624

[85] Madec Y Boufassa F Porter K Meyer L. Spontaneous Control of Viral Load and CD4 Cell Count Progression Among HIV-1 Seroconverters. AIDS (2005) 19(17):2001–7. doi: 10.1097/01.AIDS.0000194134.28135.CD 16260907

[86] Goujard C Girault I Rouzioux C Lécuroux C Deveau C Chaix M-L. HIV-1 Control After Transient Antiretroviral Treatment Initiated in Primary Infection: Role of Patient Characteristics and Effect of Therapy. Antiviral Ther (2012) 17(6):1001–9. doi: 10.3851/imp2273 22865544

[87] Saag MS Gandhi RT Hoy JF Landovitz RJ Thompson MA Sax PE. Antiretroviral Drugs for Treatment and Prevention of HIV Infection in Adults: 2020 Recommendations of the International Antiviral Society–USA Panel. JAMA (2020) 324(16):1651–69. doi: 10.1001/jama.2020.17025 PMC11017368 33052386

[88] Clark R. Sex Differences in Antiretroviral Therapy-Associated Intolerance and Adverse Events. Drug Saf (2005) 28(12):1075–83. doi: 10.2165/00002018-200528120-00003 16329711

[89] Gandhi M Aweeka F Greenblatt RM Blaschke TF. Sex Differences in Pharmacokinetics and Pharmacodynamics. Annu Rev Pharmacol Toxicol (2004) 44(1):499–523. doi: 10.1146/annurev.pharmtox.44.101802.121453 14744256

[90] Shiau S Kuhn L Strehlau R Martens L McIlleron H Meredith S. Sex Differences in Responses to Antiretroviral Treatment in South African HIV-Infected Children on Ritonavir-Boosted Lopinavir- and Nevirapine-Based Treatment. BMC Pediatr (2014) 14(1):39. doi: 10.1186/1471-2431-14-39 24521425 PMC3927631

[91] Gomes AR Souteiro P Silva CG Sousa-Pinto B Almeida F Sarmento A. Prevalence of Testosterone Deficiency in HIV-Infected Men Under Antiretroviral Therapy. BMC Infect Dis (2016) 16(1):628. doi: 10.1186/s12879-016-1892-5 27809804 PMC5096002

[92] Soldin OP Mattison DR. Sex Differences in Pharmacokinetics and Pharmacodynamics. Clin Pharmacokinet (2009) 48(3):143–57. doi: 10.2165/00003088-200948030-00001 PMC3644551 19385708

[93] Chouchana L Pariente A Pannier E Tsatsaris V Treluyer J-M. Dolutegravir and Neural Tube Defects: A New Insight. Lancet Infect Dis (2020) 20(4):405–6. doi: 10.1016/S1473-3099(20)30117-1 32222203

[94] Zash R Makhema J Shapiro RL. Neural-Tube Defects With Dolutegravir Treatment From the Time of Conception. N Engl J Med (2018) 379(10):979–81. doi: 10.1056/NEJMc1807653 PMC6550482 30037297

[95] Panel on Treatment of HIV During Pregnancy and Prevention of Perinatal Transmission. Recommendations for Use of Antiretroviral Drugs in Transmission in the United States. Available at: https://clinicalinfo.hiv.gov/en/guidelines. Accessed March 2022.

[96] Mathad JS Gupte N Balagopal A Asmuth D Hakim J Santos B. Sex-Related Differences in Inflammatory and Immune Activation Markers Before and After Combined Antiretroviral Therapy Initiation. JAIDS J Acquir Immune Defic Syndr (2016) 73(2):123–9. doi: 10.1097/QAI.0000000000001095 PMC5023467 27258230

[97] Krebs SJ Slike BM Sithinamsuwan P Allen IE Chalermchai T Tipsuk S. Sex Differences in Soluble Markers Vary Before and After the Initiation of Antiretroviral Therapy in Chronically HIV-Infected Individuals. AIDS (2016) 30(10):1533–42. doi: 10.1097/qad.0000000000001096 PMC4889571 26990631

[98] Goldstein RH Walensky RP. Where Were the Women? Gender Parity in Clinical Trials. N Engl J Med (2019) 381(26):2491–3. doi: 10.1056/NEJMp1913547 31665574

[99] Heumann CL. Biomedical Approaches to HIV Prevention in Women. Curr Infect Dis Rep (2018) 20(6):11. doi: 10.1007/s11908-018-0618-9 29666937

[100] Grant RM Lama JR Anderson PL McMahan V Liu AY Vargas L. Preexposure Chemoprophylaxis for HIV Prevention in Men Who Have Sex With Men. N Engl J Med (2010) 363(27):2587–99. doi: 10.1056/NEJMoa1011205 PMC3079639 21091279

[101] Riddell J IV Amico KR Mayer KH. HIV Preexposure Prophylaxis: A Review. JAMA (2018) 319(12):1261–8. doi: 10.1001/jama.2018.1917 29584848

[102] CDC. PrEP Effectiveness. Available at: https://www.cdc.gov/HIV/basics/prep/prep-effectiveness.html.

[103] Patterson KB Prince HA Kraft E Jenkins AJ Shaheen NJ Rooney JF. Penetration of Tenofovir and Emtricitabine in Mucosal Tissues: Implications for Prevention of HIV-1 Transmission. Sci Trans Med (2011) 3(112):112re4–re4. doi: 10.1126/scitranslmed.3003174 PMC3483088 22158861

[104] Klatt NR Cheu R Birse K Zevin AS Perner M Noël-Romas L. Vaginal Bacteria Modify HIV Tenofovir Microbicide Efficacy in African Women. Science (2017) 356(6341):938–45. doi: 10.1126/science.aai9383 28572388

[105] Marsden MD Zack JA. HIV/AIDS Eradication. Bioorg Med Chem Lett (2013) 23(14):4003–10. doi: 10.1016/j.bmcl.2013.05.032 PMC3714230 23735743

[106] Sengupta S Siliciano RF. Targeting the Latent Reservoir for HIV-1. Immunity (2018) 48(5):872–95. doi: 10.1016/j.immuni.2018.04.030 PMC6196732 29768175

[107] Chun T-W Finzi D Margolick J Chadwick K Schwartz D Siliciano RF. *In Vivo* Fate of HIV-1-Infected T Cells: Quantitative Analysis of the Transition to Stable Latency. Nat Med (1995) 1(12):1284–90. doi: 10.1038/nm1295-1284 7489410

[108] Finzi D Hermankova M Pierson T Carruth LM Buck C Chaisson RE. Identification of a Reservoir for HIV-1 in Patients on Highly Active Antiretroviral Therapy. Science (1997) 278(5341):1295–300. doi: 10.1126/science.278.5341.1295 9360927

[109] Wong JK Hezareh M Günthard HF Havlir DV Ignacio CC Spina CA. Recovery of Replication-Competent HIV Despite Prolonged Suppression of Plasma Viremia. Science (1997) 278(5341):1291–5. doi: 10.1126/science.278.5341.1291 9360926

[110] Ho Y-C Shan L Hosmane Nina N Wang J Laskey Sarah B Rosenbloom Daniel IS. Replication-Competent Noninduced Proviruses in the Latent Reservoir Increase Barrier to HIV-1 Cure. Cell (2013) 155(3):540–51. doi: 10.1016/j.cell.2013.09.020 PMC3896327 24243014

[111] Williams JP Hurst J Stöhr W Robinson N Brown H Fisher M. HIV-1 DNA Predicts Disease Progression and Post-Treatment Virological Control. eLife (2014) 3:e03821. doi: 10.7554/eLife.03821 25217531 PMC4199415

[112] Fourati S Flandre P Calin R Carcelain G Soulie C Lambert-Niclot S. Factors Associated With a Low HIV Reservoir in Patients With Prolonged Suppressive Antiretroviral Therapy. J Antimicrobial Chemother (2013) 69(3):753–6. doi: 10.1093/jac/dkt428 24187041

[113] Cuzin L Pugliese P Sauné K Allavena C Ghosn J Cottalorda J. Levels of Intracellular HIV-DNA in Patients With Suppressive Antiretroviral Therapy. AIDS (2015) 29(13)1665–71. doi: 10.1097/QAD.0000000000000723 26372277

[114] Scully EP Gandhi M Johnston R Hoh R Lockhart A Dobrowolski C. Sex-Based Differences in Human Immunodeficiency Virus Type 1 Reservoir Activity and Residual Immune Activation. J Infect Dis (2018) 219(7):1084–94. doi: 10.1093/infdis/jiy617 PMC6784502 30371873

[115] Falcinelli SD Shook-Sa BE Dewey MG Sridhar S Read J Kirchherr J. Impact of Biological Sex on Immune Activation and Frequency of the Latent HIV Reservoir During Suppressive Antiretroviral Therapy. J Infect Dis (2020) 222(11):1843–52. doi: 10.1093/infdis/jiaa298 PMC7653086 32496542

[116] Macedo AB Resop RS Martins LJ Szaniawski MA Sorensen ES Spivak AM. Influence of Biological Sex, Age, and HIV Status in an In Vitro Primary Cell Model of HIV Latency Using a CXCR4 Tropic Virus. AIDS Res Hum Retroviruses (2018) 34(9):769–77. doi: 10.1089/aid.2018.0098 PMC6152854 29926732

[117] Das B Dobrowolski C Luttge B Valadkhan S Chomont N Johnston R. Estrogen Receptor-1 is a Key Regulator of HIV-1 Latency That Imparts Gender-Specific Restrictions on the Latent Reservoir. Proc Natl Acad Sci (2018) 115(33):E7795–804. doi: 10.1073/pnas.1803468115 PMC6099847 30061382

[118] Hsu J Besien KV Glesby MJ Coletti A Pahwa SG Warshaw M. (2022). HIV-1 Remission With CCR5 Δ32 Δ32 Haplo-Cord Transplant in a US Woman: Impaact P1107, In: CROI (Conference on Retroviruses and Opportunistic Infections), 2022 February 12-16.

[119] Hütter G Nowak D Mossner M Ganepola S Müßig A Allers K. Long-Term Control of HIV by CCR5 Δ32/Δ32 Stem-Cell Transplantation. N Engl J Med (2009) 360(7):692–8. doi: 10.1056/NEJMoa0802905 19213682

[120] Gupta RK Abdul-Jawad S McCoy LE Mok HP Peppa D Salgado M. HIV-1 Remission Following Ccr5Δ32/Δ32 Haematopoietic Stem-Cell Transplantation. Nature (2019) 568(7751):244–8. doi: 10.1038/s41586-019-1027-4 PMC7275870 30836379

[121] Gupta RK Peppa D Hill AL Gálvez C Salgado M Pace M. Evidence for HIV-1 Cure After CCR5Δ32/Δ32 Allogeneic Haemopoietic Stem-Cell Transplantation 30 Months Post Analytical Treatment Interruption: A Case Report. Lancet HIV (2020) 7(5):e340–e7. doi: 10.1016/S2352-3018(20)30069-2 PMC7606918 32169158

[122] Gardner MB Luciw PA. Macaque Models of Human Infectious Disease. ILAR J (2008) 49(2):220–55. doi: 10.1093/ilar.49.2.220 PMC7108592 18323583

[123] Hatziioannou T Evans DT. Animal Models for HIV/AIDS Research. Nat Rev Microbiol (2012) 10(12):852–67. doi: 10.1038/nrmicro2911 PMC4334372 23154262

[124] Marsden MD Zack JA. Humanized Mouse Models for Human Immunodeficiency Virus Infection. Annu Rev Virol (2017) 4(1):393–412. doi: 10.1146/annurev-virology-101416-041703 28746819 PMC6542458

